# Incidence of Otolaryngological Manifestations in Individuals with Autism Spectrum Disorder: A Special Focus on Auditory Disorders

**DOI:** 10.3390/audiolres14010005

**Published:** 2024-01-04

**Authors:** Keelin McKenna, Soumil Prasad, Jaimee Cooper, Ava M. King, Shahriar Shahzeidi, Jeenu Mittal, Max Zalta, Rahul Mittal, Adrien A. Eshraghi

**Affiliations:** 1 Hearing Research and Cochlear Implant Laboratory, Department of Otolaryngology, University of Miami Miller School of Medicine, Miami, FL 33136, USA; kmcke018@med.fiu.edu (K.M.); sxp3317@med.miami.edu (S.P.); jcooper12@student.touro.edu (J.C.); amk265@med.miami.edu (A.M.K.); j.mittal@med.miami.edu (J.M.); r.mittal11@med.miami.edu (R.M.); 2 Herbert Wertheim College of Medicine, Florida International University, Miami, FL 33199, USA; 3 School of Medicine, New York Medical College, Valhalla, NY 10595, USA; 4 Grand Health Institute, Miami, FL 33132, USA; shahzeidi@yahoo.com; 5 Department of Neurological Surgery, University of Miami Miller School of Medicine, Miami, FL 33136, USA; 6 Department of Biomedical Engineering, University of Miami, Coral Gables, FL 33146, USA; 7 Department of Pediatrics, University of Miami Miller School of Medicine, Miami, FL 33136, USA

**Keywords:** sensorineural hearing loss, autism spectrum disorder, auditory deficits, sleeping problems, otitis media, sinusitis, central auditory processing disorder

## Abstract

Autism Spectrum Disorder (ASD) is a neurodevelopmental disorder characterized by stereotyped and repetitive behavior patterns. In addition to neurological and behavioral problems, individuals with ASD commonly experience otolaryngological comorbidities. Individuals with ASD often have auditory disorders including hearing loss and auditory processing disorders such as central auditory processing disorder (CAPD), as well as both chronic and recurrent otitis media. These challenges negatively impact a person’s ability to effectively communicate and may further impact their neurological functioning, particularly when not appropriately treated. Individuals diagnosed with ASD also have difficulty sleeping which contributes to increased irritability and may further aggravate the core behavioral symptoms of autism. The individuals with ASD also have a higher rate of sinusitis which contributes to the worsening of the autism behavior phenotype. The high prevalence of otolaryngological comorbidities in individuals with ASD warrants a better collaboration between their various healthcare providers and otolaryngologists with expertise in auditory, sleep, and sinus disorders in pursuit of improving the quality of life of affected individuals and their families/caregivers.

## 1. Introduction

**Figure 1:**
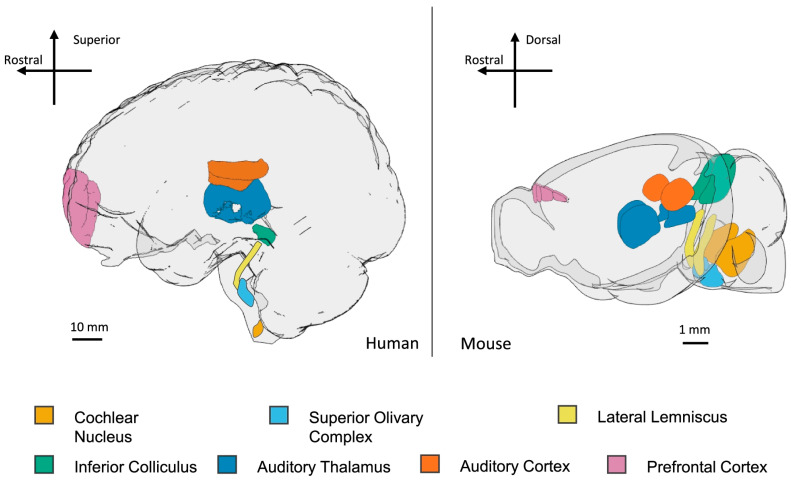
Key structures in the auditory processing pathways in human and mouse brains. Auditory information enters the central nervous system at the cochlear nuclei and is then processed at the level of the superior olivary complex, lateral lemniscus, inferior colliculus, auditory thalamus (medial geniculate nucleus and thalamic reticular nucleus), auditory cortex, and prefrontal cortex. Scale bars are approximate. Images were generated with brainrender [10] and taken from Wilde et al. [8] under a Creative Commons Attribution 4.0 International License, which permits use, sharing, adaptation, distribution, and reproduction in any medium or format, as long as appropriate credit to the original author(s) and source are given. Furthermore, individuals diagnosed with ASD have a high prevalence of recurrent otitis media [11,12]. This means an increased use of antibiotics, which can lead to higher rates of GI complications and a disturbed gut microbiome [13,14]. Finally, an increased prevalence of otitis media has been shown to correlate with a higher frequency of undergoing tympanostomy tube placement in this population [15,16]. The current incidence of ASD in the United States is 1 in 36 children, with male children being more likely to have a diagnosis [17]. Cognitive impairment is often seen in those with ASD with a prevalence between 50 and 70% [18]. Besides behavior and neurological disorders, individuals with ASD may present with comorbidities in the otolaryngology realm, such as hearing disorders, acute and chronic ear infections (otitis media), sinusitis, APDs, and sleeping problems. [15,19,20,21]. The sleep problems that children with ASD face may impact the functioning of both the individual with autism and their caretakers [22,23,24,25,26,27]. The relationship between sleeping problems and ASD shows the relevance of promptly treating sleep problems in clinical practice [22,28]. It has also been shown that individuals with ASD are more likely to have increased rates of sinusitis and have a higher number of inflammatory cells present in their nasal mucosa [29,30,31]. These issues and their association with ASD should be investigated to aid medical professionals in recognizing and promptly treating them in the autism population. The objective of this review article is to discuss the incidence of otolaryngology comorbidities in individuals with ASD (Figure 2). Better knowledge about these otolaryngology issues will lead to early intervention and better clinical outcomes.

Autism spectrum disorder (ASD) is a neurodevelopmental disorder characterized by impairments with social interaction as well as restricted and stereotyped behaviors [1,2,3,4]. Other hallmarks of ASD include a delay in speech and language development, delay in developmental milestones, and eye contact avoidance. Hyperstimulation is another pattern of behavior that is commonly observed in individuals with ASD. The individuals with ASD generally exhibit exaggerated behaviors to sensory stimuli, including hyperacusis, sensory defensiveness, aversion, and a lack of habituation to stimuli [5,6]. Hearing disorders are among the common comorbidities seen in individuals with ASD. This population has increased rates of both sensorineural hearing loss (SNHL) and conductive hearing loss [7]. With reduced receptive and expressive language abilities being a hallmark of ASD, hearing disorders may remain undiagnosed and untreated for longer in this population when compared to neurotypical people. In addition to hearing loss, people diagnosed with ASD have a high prevalence of auditory processing disorders (APDs). Although the precise etiology of APDs in ASD is not known, preclinical animal models can serve a valuable tool due to the commonality of some of the sensory structures as well as some resemblance between auditory processing pathways [8,9] (Figure 1).

## 2. Sensorineural Hearing Loss 

People with ASD have increased rates of SNHL in addition to increased rates of otitis media [5]. SNHL is prevalent in all children, with nearly 1 in every 5 children with or without ASD being affected by the age of 18; those diagnosed with ASD may be unable to communicate when hearing is suboptimal, leading to diagnostic delays [32]. The etiology of hearing loss can be congenital, traumatic, or infectious, such as otitis media. Individuals with hearing loss experience worse outcomes in their speech, cognitive function, and quality of life. Interestingly, a study investigating the prevalence of hearing loss in 8-year-old children (from 2009–2010) found that 1 in 59 children with hearing loss were also receiving services at school for autism [33]. Additionally, there was a significant difference in children with profound hearing loss having comorbid autism than those with milder forms of hearing loss. With a high prevalence of hearing loss and ASD, this is an area that requires further investigation and research. Individuals with severe hearing loss have a coexisting diagnosis of ASD at a disproportionate rate of 35.4% [33]. 

Another study evaluated 199 children and adolescents diagnosed with autism using auditory brainstem response testing. Mild to moderate hearing loss was diagnosed in 7.9% and unilateral hearing loss was diagnosed in 1.9% [34]. The prevalence of bilateral hearing loss in the ASD population was reported at 3.5%, a rate that is ten times higher than that observed in the general population. However, there was no association between the degree of intellectual functioning and hearing loss [34]. 

A systematic review covering the literature on the prevalence of hearing impairment in individuals diagnosed with ASD from 1948 to 2011 found that the diagnosis of one condition (hearing loss or autism) frequently leads to a delayed diagnosis of the other [5]. Another study reviewing 46 children diagnosed with both deafness and ASD showed that the severity of autistic behavior correlated with the level of mental deficiency, but not with the degree of hearing loss. Close to one-fifth of these children exhibited near-normal non-verbal intelligence, while another fifth experienced severe mental deficiency. Furthermore, around 23% of the children who were initially diagnosed with hearing loss did not have ASD as a diagnosis for nearly 4 years, and 22% of children with diagnosed hearing loss had an unrecognized ASD diagnosis for several years [35]. The late and previously inaccurate diagnoses also led to poorer outcomes in school. However, the hearing loss diagnosis could have contributed to a later diagnosis of ASD due to the misattribution of behaviors to hearing loss. Thus, it is recommended to test for hearing loss anytime there is a suspicion of a diagnosis of ASD. 

Autistic individuals are frequently assumed to have hearing difficulties due to unresponsiveness to auditory stimulation [36]. Paradoxically, many autistic individuals are sensitive to noise and can experience stress after hearing loud sounds, called hyperacusis. A common response for autistic patients is to cover their ears after loud stimulation [36]. The source of this increased perception was thought to be related to abnormal brain processing of sound intensity [36]. A study evaluated the subjective perception of loudness by testing the auditory dynamic range at six different tone frequencies as well as the loudness growth as a function of intensity level at 1kHz. In this study, 11 children and adolescents diagnosed with ASD were included. All the subjects had normal hearing and were free from psychotropic medication for one month before the study. There were two psychoacoustic tests administered: all pure tones were 500 ms in duration with onset/offset ramps of 50 ms, and all the stimuli were performed in one session to determine the auditory dynamic range (difference between absolute pure tone threshold). Tonal audiometry was also performed to measure pure tone thresholds at various frequencies until the subject could not hear the tone (the absolute threshold). There was also a measurement of categorical loudness scaling which was achieved by testing four ratings and one pure-tone frequency. The first phase replicated the tonal audiometry measurement at 1 kHz. The second phase involved random administrations of 1 kHz tone at different intensities in multiples of 5 dB. The subject had to rate the tone loudness as low, medium, loud, and too loud. The pure tone thresholds did not display significant differences between the groups at all frequencies. However, the autistic study group had a much lower loudness discomfort threshold level than the control group. The categorical loudness testing demonstrated that children with ASD rated the 1 kHz pure tone as “loud” at lower intensities than the control group. The group with ASD subjects also experienced a hypersensitivity to sounds of “moderate” intensity. This suggested that children with ASD had a restricted range of perception with normal absolute thresholds. Individuals with ASD are therefore likely to be irritated by everyday sounds at a higher rate than healthy subjects, suggesting hyperacusis to be a significant comorbidity in autism [36]. 

### Hearing Rehabilitation

Currently, hearing aids and cochlear implants are frequently used for therapeutic intervention for individuals with ASD and hearing loss [37]. If hearing loss is diagnosed and amplification is advised, then hearing aids are the first-line treatment. Children with ASD often experience an increased perception of loudness and may be hyperresponsive, which can affect the maximum tolerated output level of the hearing aid. Cochlear implants stimulate the auditory nerve and provide auditory information to the brain to assist with language for individuals diagnosed with profound hearing loss. They are commonly used in children, with more evidence demonstrating that they can help with speech and language development. Cochlear implants have also shown an improvement in social communication, eye contact, and environmental awareness in individuals with ASD [5,38]. Another representative study of young children who received cochlear implants revealed that those with ASD showed improvements in receptive and expressive language at a rate that was half of what was observed in children without a concurrent ASD diagnosis [18]. The long-term speech recognition abilities after a cochlear implant in children with complex developmental issues is related to their developmental profile, which should be considered for counseling. Cochlear implants appear to show a generally positive outcome in speech and language development through auditory stimulation. Cochlear implants are the only effective treatment for moderate to profound SNHL when amplification does not provide adequate access to spoken language and can also improve child–caregiver engagement. 

A recent retrospective case review and parent survey were conducted to review outcomes of cochlear implantation in children with ASD at a tertiary care children’s hospital. The interventions were either unilateral or bilateral cochlear implants given to 30 children with ASD who underwent implantation between 1991 and 2018. All the children had full electrode insertions with no cochlear nerve deficiency. A total of 68% were male participants, 47% had unilateral implants, and 16% received bilateral simultaneous implants. The average age for the first implant was 3.5 years, and the average age of ASD diagnosis was 5.1 years. Electronic medical records were obtained and reviewed for electrode insertion, etiology of hearing loss, age at diagnosis, speech perception tests, and communication modes. A parental survey of behavior was also sent after the implant. Around 33% of all implanted individuals, and 45% of consistent device users developed open-set speech perception after an average of 4.5 years of device use. Auditory integration scores were measurable for 15 children and improved for 7 of them. One child with significant cognitive impairment had no measurable auditory improvement and six children had no change in their scores while improving auditory thresholds. Parent reporting to the audiologist demonstrated that 86% of children showed an improvement in social engagement compared to the children before having a cochlear implant; however, the low sample size should be recognized. There was high variability in the ranking of affected behavior. Emotional needs of the child had the highest average ranking, while awareness of the environment had the lowest average ranking. These findings suggest that although there are no methods present to accurately predict future cognitive abilities with implantation, early intervention can likely change the trajectory of development. The improved hearing and understanding of a spoken language may improve cognitive function, especially for those with ASD [5]. This study had the longest follow-up reported to date, demonstrating the speech perception and language skills have benefits that can enhance childcare and development. There has been a delayed emergence of skills seen in implanted individuals. Speech perception and speech expression are two of the predominately impacted areas of functioning when a person receives a cochlear implant. In children diagnosed with ASD, it has been shown that there is significant improvement in both of these categories. The timing of the first implant can also affect the development of open-set speech perception. The most improvement is seen when implants are placed before 3 years of age. However, 12 of the 30 subjects were implanted at ages over 3 years old, making comparisons of expected rates of speech perception development difficult, even if the rates were lower than a normal cochlear implant population. Around 13 of the 30 subjects used spoken language to some degree and 4 used oral communicators with mainstream educational placement. The standard audiologic measures may not be able to analyze the improvement in the quality of life; however, the study strongly suggests that improved social engagement was perceived by the parents. 

Eshraghi et al. studied the improvement in speech expression and speech perception in children with ASD who received cochlear implants [38]. Both speech expression and perception were rated on a scale of 0–4, with 0 being no vocalization or awareness of the environment, and 4 meaning they can produce sentences and understand conversations, respectively (Table 1). 

Preoperatively, 12 out of 14 children in the autism group were in the lowest category (0–1). Postoperatively, 67% of those in the ASD group were in category 3 or 4 for speech perception and 33% were in category 1. Speech expression data were similar, with 93% of the ASD group being category 1 preoperatively. Postoperatively, 60% of participants from the ASD group improved to a category 3 or 4. The improvements seen in the ASD cohort are functionally significant, although the result is not close to what is observed in neurotypical kids but instead, is similar to the improvement seen in the neurotypical controls in this study. Through a parental survey, they also found that parents reported the greatest improvements in name recognition, response to verbal requests, and enjoyment of music [38]. Cochlear implantation is a viable option for some children with ASD to improve auditory skills, language skills, and overall social functioning. 

Otolaryngologists and audiologists should be cautious and follow clinical guidelines when making a diagnosis and plan of care for SNHL in individuals with ASD, while also recognizing the importance in the timing of a cochlear implant or other therapy. A novel grading system is also proposed that allows researchers to better assess the functional speech perception and speech expression post-cochlear implantation (Table 1). This novel system allows us to appreciate the benefit of implantation in this group with a limited potential for speech development even without hearing loss. It should be noted that wearing hearing aids or the external portion of a CI may be initially challenging for some autistic individuals with sensory disorders. This challenge can benefit from the support of applied behavior analysis (ABA) therapy.

## 3. Tinnitus and Misophonia

Tinnitus is the conscious perception of an auditory sensation without any external stimulus, and hyperacusis is a hypersensitivity to sound. Both of these can be highly distressing to individuals with autism, as they can be a constant source of sensory input regardless of the external environment. Danesh et al. found that hyperacusis and tinnitus are more prevalent in individuals with ASD, specifically in those diagnosed with Asperger’s syndrome [39]. The diagnosis of Asperger’s syndrome is no longer recognized as a clinical diagnosis, and these individuals with less severe ASD are currently recognized under the umbrella term ASD. In persons with a decreased tolerance for sensory input such as some individuals diagnosed with ASD, both of these conditions can cause increased irritability as well as negative behavioral changes.

Misophonia is another auditory disorder found among individuals with a decreased sound tolerance, such as those with ASD. It is a condition where specific sounds elicit inappropriate behavioral and emotional responses. The triggering sounds usually are human-made and repetitive, such as chewing, sniffing, and tapping. The response to these noises varies in intensity, but is most often a response of annoyance and anger. In individuals with ASD, this is among the commonly encountered disorders dealing with a decreased sound tolerance along with hyperacusis and phonophobia. This innate reaction to specific sounds can contribute to the behavioral and emotional liability that is a hallmark of ASD [40]. Misophonia has not been as extensively investigated as other sound-processing disorders mentioned above. There are studies indicating the neural pathways that may be involved in the behavioral responses seen in misophonia, which include the limbic system, non-classical auditory pathway, and the auditory cortices [41]. There is also some evidence that psychotherapy (cognitive behavioral therapy) alone or in conjunction with therapies such as sound exposure may be helpful in the management of misophonia [42]. In children in an educational setting, individualized education plans (IEP’s) can be instrumental in allowing a child affected by misophonia to learn and develop at school. The investigation of the precise links between ASD and misophonia should be conducted to both understand and treat this distressing comorbidity of ASD. 

## 4. Auditory Processing Disorders in ASD

**Figure 2:**
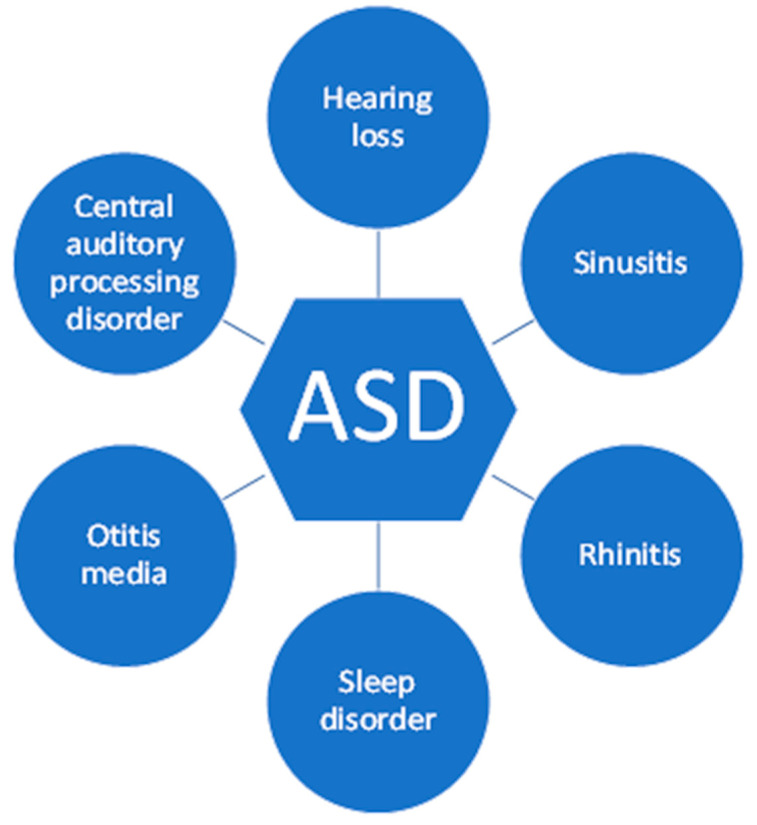
A schematic representation of otolaryngological complications in individuals diagnosed with autism spectrum disorder (ASD).

APDs such as central auditory processing disorder (CAPD) are common in those with learning disabilities, such as ASD and ADHD, and often initially present in children. Central auditory processing (CAP) is achieved through the central auditory nervous system by the conduction of auditory action potentials following a stimulus [9,43]. When this is disrupted, individuals have difficulty with sound and speech perception, especially in noisy environments, as well as trouble learning and responding to others verbally. The key structures in the auditory processing pathway in human and mouse brains are shown in Figure 2. APDs and ASD share a complex relationship that has yet to be fully understood. The evidence on APDs in this population is mixed, there is evidence suggesting an impairment in auditory processing in this population, but also evidence that they have enhanced processing.

In a systematic review, Gonçalves et al. looked at the relationship between ASD and APDs [44]. This study found that CAPD has cortical and subcortical portions. Brainstem auditory evoked potential (BAEP) measured the subcortical component and was used in multiple studies to evaluate ASD risk. Changes in BAEP can be indicative of APDs, as shown by absolute and interpeak latency changes. The cortical component was measured using the N1 wave response, which showed prolonged latency. The findings of multiple studies further strengthened the connection between ASD and CAPD. One of the studies included in the above systematic review was by Azouz et al., who conducted a study in Egypt to demonstrate the relationship between CAPD and ASD [45]. A total of 30 children with ASD from the Alexandria University Children’s Hospital in El Shatby were enrolled. In-depth medical histories were collected from each participant followed by a neurologic evaluation. The Stanford–Binet test determined the intelligence quotient (IQ) of the participants. Multiple tests were conducted to assess hearing and communication. First, to assess hearing, the auditory brainstem response (ABR) was tested. Then, pure tone audiometry and impedancemetry tests were performed in cooperative subjects. Next, to assess communication skills, researchers used the test of acquired communication skills. In addition, an electrophysical evaluation and the sensory checklist for auditory/listening skills were completed. These data were looked at against data from 15 children with normal hearing. When compared to the normal group, the ABR tests showed prolonged latencies in both the right and left ears. In general, N1c amplitude is indicative of secondary auditory cortex activation. Azouz et al. found that this amplitude was flipped between the neurotypical children and those with ASD and concluded that auditory stimuli are processed more in the right hemisphere in autistic children compared to neurotypical participants [46,47]. N1c amplitude was found to be lower, and the absolute latency of N1c was prolonged in the ASD group. The specific prolonged ABR latencies show that the brainstem may be involved, in ASD this could lead to issues with language and cognition. Overall, the results of this study constitute abnormal auditory responses in the ASD group, thus highlighting the pathological role of CAPD in ASD [45,46,47]. An impaired lateralization, presenting as increased right hemispheric or decreased left hemispheric activity, is thought to play a role in language processing disorders in people with ASD [48]. Decreased left-sided cortical activity has been seen with decreased left temporal and left insular activity in individuals with ASD [48]. Differences in hemispheric lateralization means that people with ASD have more brain symmetry when compared to the left hemisphere dominance in neurotypical individuals. The increased right hemispheric activity is thought to be secondary to impaired neurodevelopment but may instead be secondary to other causes.

Feig et al. also emphasized that CAPD is a pillar of ASD [49]. In this study, there were 53 participants: 26 children with ASD and 27 typically developing (TD) children. There were two main parts to this study. First, there were tasks that evaluated the visual and auditory gap detection thresholds, done on a computer. Gaps in the presented stimuli were randomized, and participants decided which intervals had gaps. Second, language assessments looked into language and communication difficulties in children with ASD. The Comprehensive Test of Phonological Processing (CTOPP), which focuses on phonology, and the Clinical Evaluation of Language Fundamentals, fourth edition (CELF-4), which focuses on language comprehension, were used. The study found that children with ASD need longer gaps in order to determine gap presence reliably in the auditory versions of the test. However, with visual gaps, there was no significant difference between ASD and TD groups for determining the presence of a gap. Results from the language assessments demonstrated a direct positive relationship between phonological memory and temporal auditory resolution. A negative trend was seen between gap detection and receptive language function, meaning that a worse gap detection in the ASD group may be associated with worse language skills. These results suggest that communication problems in children with ASD could be related to subpar auditory gap detection. Visual processing results were not significant. Future studies should take a deeper dive into the relationship between language comprehension and function and auditory processing [49]. 

The mechanism of impaired auditory processing in individuals with ASD is still unknown. One study looked at preconscious automatic auditory processing with electrophysiology using a mismatch negativity paradigm (MMN) [50]. The MMN is used to demonstrate automatic orienting reflexes after detecting disturbances in an individual’s direct environment. For example, an occasional outlier sound can be played after a series of repeating standard sounds, which then causes a negative deflection in the electroencephalography (EEG). A positive deflection, called P3a amplitude, can also be studied. The deflection on the EEG will usually occur 100–200 ms after the stimulus. MMN has been used to study schizophrenia and ASD, which have been found to have a clinical and biological overlap [50]. However, previous results in the MMN for individuals with ASD have been highly inconsistent. The study investigated the association between ASD and P3a amplitude using the MMN. Subjects were seated in an armchair in a sound-insulated room next to a control room and instructed to watch a movie and ignore the sounds that were presented. There were four stimuli at different frequencies with three deviant stimuli presented. There was a positive correlation between the frequency MMN and P3a and the duration MMN for the whole group. The MMN also reached a maximum amplitude at the frontocentral region of the brain; however, for the subjects with ASD, there were MMN amplitudes triggered by duration and frequency–duration deviants. The smaller MMN amplitude in ASD children suggested that there was a less accurate automatic orienting reflex to deviancy. A smaller amplitude indicates hyporesponsiveness to sensory stimuli, which has been hypothesized to be associated with ASD children. The smaller amplitude may be suggestive of a deficiency in temporal auditory processing or temporal discrimination. Interestingly, there was an association between MMN amplitude and the severity of behavioral problems experienced by individuals with ASD. Thus, children with ASD might be less responsive to environmental stimuli on both sensory and attentional levels [50]. 

Another study utilized magnetoencephalography to compare cortical responses between ASD and TD individuals to the passive mismatch paradigm [51]. The subjects completed the paradigm in a quiet and noisy environment and the mismatch field response was obtained. Within the quiet condition, there were common neural sources of mismatched field responses in both groups, at the right temporal gyrus and inferior frontal gyrus. However, in the noisy condition, the mismatch field response in the right inferior frontal gyrus was preserved in the TD group and reduced in the ASD group. The response of the mismatch field was correlated with the severity of ASD. Thus, the recruitment of neural resources in ASD with a reduction in top-down modulation is needed to decrease the impact of noise on auditory processing [51]. A different team investigated the auditory evoked field (AEF) synchronization in ASD subjects. They used the Omega complexity analysis to determine the global coordination of AEFs in 3- to 8-year-old TD children. AEF is a brain response to auditory stimuli recorded by magnetoencephalography and is the equivalent of auditory evoked potential recorded by electroencephalography [52]. They found that children with ASD had significantly higher Omega complexities in the time window of 0–50 ms, suggesting lower whole-brain synchronization in the early stage of the P1m component. There were no significant differences between TD and children with ASD in any time window. These results were consistent with reports of reduced brain hemodynamic synchronization in individuals with ASD. The reduced interhemispheric connectivity may cause reduced synchronization during auditory information processing [51]. 

Previous literature has looked into the decreased activation of left speech-related temporal areas in adults with autism while listening to speech-like sounds [53]. A research team investigated if there was abnormal cortical processing in children with ASD. This was achieved through a regional cerebral blood flow measurement with PET after giving medication. The subjects included 11 children with ASD and 6 nonautistic intellectually disabled children evaluated during rest. The study found that there was significantly less activation in the autistic group localized to the left speech-related areas. Thus, this activation study performed in children with ASD confirmed previous findings for adults. This impaired processing could be involved with the behavioral responses to sounds that are characteristic of individuals with ASD [53]. 

Goncalves et al. executed a systematic review to understand auditory sensory alterations in individuals with ASD. They analyzed questionnaires, imaging, and behavioral and electrophysiologic studies to gain insight on this topic. ABR studies showed that a portion of the cohort had longer latency, although this was not consistent across all stages of development, and EEGs showed unstable neural tracking in subcortical auditory processing [54]. They showed that those with ASD had an improved auditory perceptual capacity in conjunction with a reduced auditory discrimination and detection [54]. The deficits that were seen seemed to be correlated with the severity of language impairment as well as social impairment. These findings were inconsistent across the population and across all ages; they suggest the utility of longitudinal studies to better understand these changes over time or between various patient subgroups. Key et al. found that individuals with ASD had relatively intact neural responses to single speech sounds, but had difficulty with categorical speech discrimination and processing of semantic content [55]. As shown by Goncalves et al., these deficits were more prominent in younger individuals. Chen et al. found that in the ASD population, there was an enhanced pitch perception, although this was also dependent on language and communication abilities and age [56]. The commonality with these studies underlies the heterogenicity of this population with regard to auditory processing disorders, particularly in relation to linguistic abilities and age [54,55,56,57]. Higher level processing is a multifaceted process that, as these studies show, is not uniformly impacted across the spectrum of autism. There are aspects of auditory processing that are impaired, while others may be enhanced in individuals with ASD when compared to neurotypicals. As suggested in the study of Chen et al. [56], future studies employing neurophysiological and brain imaging techniques, particularly those with a longitudinal approach, are essential for a deeper understanding of the neural mechanisms underlying atypical pitch processing in individuals with ASD. The information derived from these studies will be instrumental in informing the development of auditory-based interventions aimed at enhancing language and social functioning. In addition, it is possible that the communication challenges faced by individuals with ASD may be more closely linked to a diminished interest in social interaction rather than fundamental auditory impairments, which needs to be explored in future studies.

It is essential for clinicians to recognize that APDs can impact an individual’s functioning beyond language expression and reception, with impacts on behavioral and emotional functioning. The recognition of APDs in children with ASD helps clinicians to appropriately counsel caregivers in the best practices in school and work environments. Behavioral tests for CAPD should be further developed, as they are currently difficult to evaluate due to their subjectivity and complexity [44]. Due to the nature of ASD, behavioral tests may be difficult or unreliable in this population. The development of novel testing for APD utilizing electrophysiology has been an emerging area. Liu et al. conducted a systematic review and found that there are seven testing modalities that rely on electrophysiology currently, including click-evoked auditory brainstem responses, frequency-following responses, the binaural interaction component of the auditory brainstem responses, middle-latency response, cortical auditory evoked potential, mismatch negativity, and P300 [58]. They found that all of these tests have the potential to be used for the diagnosis of APD, but more research is warranted to completely understand the results of the tests [58]. The development and implementation of standardized and objective electrophysiologic techniques for the diagnosis of APD is crucial to better help this population. The utility of current treatments for APD such as environmental modifications (hearing assistive technology, preferential seating, and written instructions), speech therapy, and auditory training such as the FastForWord program, should be evaluated in this population. Emerging evidence indicates that diet and the gut microbiome may also be factors in APDs [59]. Probiotics and diet alterations should be evaluated as treatment modalities for processing disorders in the ASD population, as they may decrease inflammation and modify neural connectivity [59].

## 5. Balance and Vestibular Dysfunction

Individuals with ASD may be affected with balance issues and vestibular dysfunction. The human vestibular system is located encased in bone in each temporal bone, located near the cochlea. This works in conjunction with the cerebellum and eyes to maintain balance and react to changes in the environment. There are studies that cite cerebellar differences in individuals with ASD, such as decreased cerebellar volume and decreased density of Purkinje cells, while others have refuted this [60]. Individuals with ASD are known to have postural instability, balance issues, and abnormal responses to vestibular stimulus [61,62]. Historically, these issues were thought to be related to poor motor control, while more recently, there is emerging evidence that these issues may be related to vestibular dysfunction. Administering the appropriate tests to not only identify motor control disorders but also vestibular disorders is imperative; these include video-nystagmography (VNG), caloric testing, and vestibular evoked myogenic potential (VEMP) as tolerated in the population.

Therapeutic interventions for vestibular dysfunction in individuals with ASD primarily focus on testing and strengthening the neuromusculature performing balance control. Dysfunction can manifest as dynamic or static postural issues, leading to higher velocity and more exaggerated swaying. Sensory integration (SI) therapies target an individual’s coordination, muscle strength, and speed as a means to train the dysfunctional vestibular system [62]. Various SI therapies are currently used in clinical practice including swimming, trampolines, martial arts, transcranial direct current stimulation, animal-assisted therapies such as riding, sports, dance, videogames, as well as specific balance training exercises. It has been found that these therapies can enhance an individual’s sensory input integration processes, motor skills and coordination, and cerebellar function, which all help to improve balance control [63]. SI therapy testing can also be used as a diagnostic tool for clinicians in the early detection of ASD and vestibular dysfunction; however, auditory dysfunction is sometimes wrongly attributed to other neurodevelopmental disorders [64]. Additionally, the most successful SI therapies with the greatest adherence to the programs were those that the ASD individual actively enjoyed. Hence, therapeutic efforts should focus on the treatments with the largest continued interest and utilization from ASD patients and should collect metrics on the participants’ agreeableness to the intervention. It is important to note that the majority of SI therapies have been designed specifically for children with ASD. Since ASD is chronic, and therefore, present in adults, the current SI therapies should be tested and adapted for older ASD populations to also improve their quality of life. An elevated awareness of vestibular dysfunction in ASD individuals is essential to enhance the clinical management of this distinct patient group and advance their overall clinical outcomes.

## 6. Suppurative and Serous Otitis Media

### 6.1. Increased Risk

There is some evidence that individuals with ASD may have increased rates of both serous otitis media and suppurative otitis media, with the latter leading to increased antibiotic use in this population. Sabourin et al. conducted a case–control study in the United States to determine if children diagnosed with autism had a greater risk of infection, including ear infections, compared to other developmental disorders and healthy controls [11]. The children were aged 30–69 months and were born between 2003 and 2006. The children with autism were enrolled from educational and clinical settings, and the controls were randomly drawn from birth certificates. Neonatal infections, those that happened in the first 28 days of life, were assessed through medical records. Infections before the age of 3 were assessed through a caregiver interview. The study found that children with ASD had significantly higher odds of having a neonatal infection, infection by 1 year of age, and infection by 3 years of age compared with the healthy controls. Children with ASD were also at higher odds of having a neonatal infection compared with children with other developmental disorders. Additionally, children with autism who experienced regression or loss of language or social skills for at least 3 months were at higher odds of having an infection by 1 year of age. This suggests that infections in childhood, including ear infections, may be associated with the symptoms of autism, such as difficulty with language and social skills [11]. Suffering from infections, of any etiology, can be detrimental to an individual and their care team’s routine and functioning, particularly when they have a diagnosis of ASD [65,66]. 

A study by Davignon et al. assessed the prevalence of ear infections, as well as various other medical and psychiatric conditions, and their association with ASD in adolescents and young adults [12]. Participants aged 14 to 25 were recruited from the Kaiser Permanente Northern California health system in the United States. Individuals diagnosed with ASD were compared with individuals with ADHD, diabetes mellitus, and typical controls. The study found that the most frequently reported medical condition by individuals with ASD was infection (42%). This included ear, GI, skin, and upper and lower respiratory infections. Overall, co-occurring medical conditions were more common in individuals with ASD than those with ADHD or typical controls. Otitis media, both suppurative and serous, were specifically prevalent in autistic participants. It was observed that 5.8% of participants had a history of ear infections compared to 3.0%, 4.6%, and 4.4% in typical controls, participants with ADHD, and participants with diabetes mellitus, respectively. Participants with ASD had significantly higher odds of having an ear infection compared to typical controls (1.70) and those with ADHD (1.26). The odds ratio comparing participants with autism to those with diabetes mellitus was insignificant. This study demonstrates that adolescents and young adults with autism are at an increased risk of having infections, specifically ear infections, and may require more medical attention in this area [12,34]. The research on OM and ASD primarily consists of small-scale studies. Given this limitation, there is a need for larger studies to gain a clearer understanding of the prevalence and outcomes related to this potential association. While there is some evidence suggesting an increased risk of OM and potential hearing-related challenges in individuals with ASD, more research is needed to fully understand the nature and extent of these associations. 

### 6.2. Antibiotic Association

Wimberley et al. performed a study using 780,547 Danish children to assess the association between ASD, otitis media, and antibiotic use [67]. The participants were born between 1 January 1997 and 31 December 2008 and were followed until 31 December 2012. Data were collected using the Danish National Patient Register, prescription redemption data, and the Danish Psychiatric Central Research Register. Over the course of the study, 9352 children developed autism, 43,149 were diagnosed with otitis media, and 49,713 used a broad-spectrum antibiotic. The median ages for the first otitis media diagnosis, antibiotic use, and autism diagnosis were 1.5, 5.4, and 7.1 years, respectively. Male participants were more likely to be diagnosed with both otitis media and autism but used fewer antibiotics. A statistical analysis found that children who were diagnosed with otitis media (aHR = 1.83) or who used broad-spectrum antibiotics (aHR = 1.29) were at increased risk of being diagnosed with autism. The exposure to other infections increased the risk of autism (aHR = 1.23) but not as significantly as otitis media (aHR = 1.96). This study demonstrates that otitis media and antibiotic use are associated with the risk of developing symptoms of autism that needs to be explored in future studies [67]. 

Bittker and Bell also looked at the association with antibiotics by performing an epidemiological study in the USA, investigating the risks of autism associated with the postnatal environment [14]. The objective of the study was to determine if acetaminophen use, antibiotic use, incidence of ear infection, shorter period of breastfeeding, or decreased use of oral vitamin D drops were associated with the diagnosis of ASD. The researchers provided Qualtrics surveys to biological parents of children aged 3 to 12 years, assessing for exposure to any of these variables. A total of 1001 children had a diagnosis of autism, and 514 children did not. The study found that acetaminophen (*p* = 0.026), antibiotics (*p* < 0.001), ear infections (*p* = 0.003), and a decreased duration of breastfeeding (*p* < 0.001) were all associated with an increased incidence of autism. A decreased use of oral vitamin D drops was only weakly associated (*p* = 0.102). Since antibiotics are often used as the treatment for ear infections, these variables were studied more closely to determine if both are potentially causative or if one is correlated because of the other. When looking at a model for antibiotic use and ear infections, they found that antibiotic use was the stronger variable (*p* = 0.0322), and ear infections were insignificant (*p* = 0.3546). This suggests that an increased incidence of ear infections may be associated with an increased risk of autism due to the more frequent treatment with antibiotics. The increased use of antibiotics may impact a person’s gut microbiome, affecting the gut-brain axis and hence, increasing their predisposition to ASD [14]. 

Alshammari et al. also discussed the association between otitis media and antibiotic use and its potential association with GI disturbance [13]. They conducted a trial in Saudi Arabia to assess the incidence of *Clostridium perfringens* in individuals diagnosed with ASD and evaluated the bacterial susceptibility to current treatments. The study consisted of children aged 3 to 12 years old. A total of 57 had a diagnosis of autism, and 57 were healthy controls. They were subdivided into groups based on the presence or absence of a diagnosis of autism and whether or not they had gastrointestinal symptoms. Fecal samples were obtained from each participant and were cultured for *C. perfringens*. The study found that 38.6% of fecal samples taken from autistic participants had significantly higher levels of *C. perfringens* compared to the controls (*p* = 0.003). The highest incidence was in autistic participants who also presented with GI symptoms (*p* = 0.001). *C. perfringens* has been shown to induce GI complications and could be associated with symptoms of autism, including rigid behaviors and unusual sleeping and eating habits. Alshammari et al. discusses that the overuse of antibiotics in treating recurrent otitis media may lead to an overgrowth of this harmful bacteria and produce a general gut dysbiosis that may contribute to GI problems. This, in turn, can contribute to the behavior changes seen in ASD [13]. However, further studies are warranted to precisely decipher the causative link between antibiotic use, the gut microbiome, and a predisposition to ASD, which will provide novel avenues of prevention and treatment.

### 6.3. Effect on Behavior

Cohen and Tsiouris performed a large-scale study including 2243 participants in order to investigate how certain factors, including otitis media, may affect aggressive behaviors in individuals with autism or intellectual disability [68]. Triggers for aggressive behaviors were studied in relation to intellectual disability, neuropsychiatric diagnoses, demographics and ongoing medical conditions. Of note, the study found that ear infections were associated with an increased verbal (*p* = 0.017) and physical (*p* = 0.001) aggression towards themselves. Aggression against others was insignificant. Participants with ear infections (*p* = 0.013) or with a diagnosis of ASD (*p* = 0.007) were more likely to have aggressive behaviors triggered by frustration. Discomfort was a trigger for aggression in those with ear infections (*p* = 0.024) and those with ASD (*p* = 0.026), particularly autistic female participant. Additionally, change was likely to be a trigger for aggression in participants diagnosed with ASD (*p* = 0.001). These results suggest that medical conditions, such as ear infections, and diagnoses, such as ASD, can affect the stimuli that trigger aggressive behaviors. The authors suggest that the self-injurious behavior noted in the study may be learned through an operant conditioning model. Physiologic pain may increase the arousal of the individual, leading to behaviors such as self-injury. The self-injurious model is maintained when subsequent unpleasant physiologic stimuli are felt. The pain from an ear infection may be one of the necessary stimuli to trigger this sort of behavior. Individuals with ASD are more likely to struggle with this, as they are not always able to recognize or verbalize their discomfort. Early recognition and treatment of these ear infections may help to diminish some of these harmful behaviors [68]. 

Additionally, Myne and Kennedy performed a retrospective study in the United Kingdom on 61 children who presented to pediatric audiology with hyperacusis, which they defined as an increased sound sensitivity that interfered with the child’s behavior or daily life [69]. They used case notes to assess the presentation in the clinic and comorbid conditions. The data collection included variables such as age, gender, symptoms, troublesome sounds and the child’s reaction to them, and associated medical conditions. They also assessed for the presence of conductive or SNHL and otitis media. The study found that an active middle ear problem was present in 48% of the children. Otitis media with effusion was the most common at 38%. Five other children had a history of otitis media for which they had previously been treated. Neurodevelopmental disorders were found in 46% of the participants; ASD was the most common at 13%. Individuals with ASD are known to have inappropriate sound processing and are less tolerant of loud sounds as discussed previously. The addition of otitis media could aggravate this situation further, manifesting in behavior changes such as covering the ears, screaming, or hiding [69]. 

### 6.4. Disparities in Surgical Management

Due to increased rates of otitis media, individuals diagnosed with autism are also more likely to undergo a tympanostomy tube (TT) placement. Yan et al. evaluated data in the United States from the National Health Interview Survey from 2014 to investigate the frequency of TT placement in individuals with ASD [16]. This surgical procedure is extremely common among the pediatric population and is used in the treatment of unilateral or bilateral otitis media with effusion. A total of 11,370 children under the age of 18 were included, 239 of which were diagnosed with ASD. The study found that children with ASD (14.2%) were significantly more likely to undergo TT placement compared to neurotypical children (8.6%), with a p-value of 0.002. Children with autism were also at increased odds of getting a TT placed (OR = 1.52). Additionally, increased odds of TT placement were associated with the male sex, white race, and non-Hispanic ethnicity. The authors list several explanations for the increased prevalence in autistic children including an increased rate of otologic disease, perceived heightened benefit for TT placement, over-diagnosis of ear disease due to difficulty in examining the patient, and overestimation of hearing loss in children with communication difficulty. They also suggest that overdiagnosis of otitis media may be due to the fact that children with autism are much more likely to scream or cry during an examination, causing the eardrum to appear red, mimicking a bacterial infection. Additionally, traditional audiometry requires that the patient has an appropriate reaction in response to the cue, which may be difficult in children with autism who have abnormal responsiveness to auditory stimuli or processing difficulties. Finally, both clinicians and parents may be quick to intervene surgically in an attempt to aid in the development of language. Although it is possible that otitis media is more common in children with autism, care should be taken by both otolaryngologists and audiologists to make sure that the diagnosis and TT placement are based on sound clinical guidelines [16]. Adams et al. also found that children diagnosed with ASD were more likely to undergo tube placement [15]. They performed a retrospective case cohort study to evaluate the prevalence of otitis media and the frequency with which these individuals underwent pressure equalizer (PE) tube placement. A total of 48,762 individuals with a diagnosis of ASD were matched with 243,810 neurotypical controls. These children were 2–18 years of age. They found that the individuals diagnosed with ASD were significantly more likely to undergo PE tube placement than matched controls, and this was consistent across all age groups. Interestingly, they also identified that male participants made up a larger percentage of the patient population with ASD (80%). However, female participants were more likely to undergo PE tube placement compared to controls. PE tubes were also placed significantly earlier in individuals with ASD, although the actual difference in the age was small. The authors suggest that this disparity may occur due to the co-occurring language impairment in this patient population, which may make it difficult for providers to appropriately identify the proper disease process and utilize the appropriate management in these children. This stresses the importance of accurately performing an ear exam in individuals diagnosed with ASD so the best management plan may be made [15]. 

When planning a surgical procedure for individuals diagnosed with ASD, careful consideration must be used when choosing anesthetic agents. Individuals with ASD are known to be under greater oxidative stress at baseline than their neurotypical counterparts due to alterations in their redox mechanisms; they are also known to have mitochondrial dysfunction [70,71]. Nitrous oxide is a commonly used anesthetic agent that can induce oxidative stress, which can harm those with a diagnosis of ASD. It does this through the depletion of the vitamin B12/folate system and deactivation of methionine synthase, leading to more oxidative stress and excitation of NMDA glutamate receptors. This is especially concerning in those with an MTFR deficiency or those with a B12 deficiency, as many individuals with ASD have [72]. Care must be taken by all hospital staff interacting with a person who is diagnosed with autism, as a surgical center can be an overstimulating and stressful place for them. Alternate routes of premedication (IM vs. oral) may be necessary to ensure the person receives a proper dose. This will help ensure that the patient has the safest and least stressful experience while at the hospital and will help the hospital staff to efficiently complete the surgery with the appropriate perioperative management of this vulnerable population.

## 7. Sinus Disorders

Although there are limited data on the association between ASD and sinus conditions, some data have shown that individuals with ASD are at increased risk of having sinusitis. Koceski and Trajkovski analyzed data from Macedonia to better understand the health status of individuals with autism compared to neurotypical controls [29]. The study population consisted of 72 individuals with a diagnosis of ASD and 75 healthy controls between the ages of 3 and 24. They utilized the Rochester IV health status determination form, which is used to evaluate factors such as the presence of medical conditions, functional ability, and use of healthcare services. Interviews were conducted with the parents, guardians, or caregivers. The study found that certain respiratory conditions were more common in participants with ASD, including rhinitis/sinusitis. A total of 40.3% of participants with ASD had a history of sinusitis, while sinusitis was present in only 17.3% of controls (*p* = 0.02). Sinusitis also seemed to correlate with increased rates of acute otitis media. The early recognition and treatment of this disease could decrease discomfort in individuals with ASD, thereby improving their behaviors, communication, and importantly, their quality of life [29]. 

Harville et al. also found increased rates of sinusitis in individuals with ASD [30]. They performed a study in the United States to determine if individuals diagnosed with autism were more likely to express HLA-Cw7 than neurotypical controls. Human leukocyte antigens (HLA) help to shape the immune system and are known to be implicated in many disease processes. Cell lines were obtained from 126 individuals with ASD, along with 17 lymphoblastoid cell lines taken from Autism Genetic Resource Exchange and the National Institute of Mental Health. HLA typing was completed on each of the cell lines to determine the presence of HLA-Cw7. The results were then matched against a national database. HLA-Cw7 was more prevalent in the ASD group, especially in Caucasians (*p* < 0.001). The allele was present at twice the rate compared to the rest of the population. The study also analyzed the presence of comorbid conditions and their association with the HLA-Cw7 allele. Participants that harbored this allele were more likely to have increased immune system activation, including chronic sinusitis. The authors suggest that HLA dysfunction may lead to a chronic inflammatory state, thereby inducing chronic sinusitis [30]. 

In addition to a higher prevalence of sinusitis, it has been shown that individuals with ASD have an increased number of inflammatory cells present in the nasal mucosa. Caruso et al. performed a study to better understand nasal cytology [31]. The objective was to identify the presence of inflammatory cells (eosinophils, mast cells, and lymphocytes) in the nasal passages of children diagnosed with ASD. Neutrophils were excluded because they are known to exist as “scavengers” in normal mucosa. A total of 150 children were included, 85 male participants and 65 female participants who had a diagnosis of ASD, who were then paired with neurotypical controls. The specimen collection was achieved by performing a nasal scraping on the mucosa located in the middle part of the inferior turbinate. Glass slides were prepared and then reviewed under a light microscope. It was discovered that children with ASD had a significantly higher amount of inflammatory cells present in their nasal mucosa. They found that in children with ASD, 25% of the cells were eosinophils, 20% were lymphocytes, and 20% were mast cells, compared to 5%, 5%, and 1%, respectively, in controls. More research on this subject must be done to better understand the pathologic process related to this finding, but this may allow us to better understand how the immune system affects the neurologic development of individuals with ASD [31]. 

## 8. Autism and Sleep Disorders

Anywhere from 40 to 83% of individuals with ASD have some form of sleep disturbance [73,74,75,76,77,78,79]. In young children not yet diagnosed with ASD, sleep problems are among the first concerns that parents report, and this tends to persist through adulthood [80,81,82]. Sleep disorders are highly prevalent but not universal in this population [75,82,83]. Autism is a diverse condition with a noted heterogeneity, with sleep disorders reflecting this diversity. Sleep problems in ASD can include insomnia, parasomnia, sleep-disordered breathing, circadian rhythm disorders, daytime sleepiness, and/or sleep movement-related disorders [76,77,79,84,85,86]. As in the general population, individuals with ASD most frequently experience insomnia as their primary sleep issue. This often manifests as extended periods needed to fall asleep, prolonged awakenings during the night, and waking up early in the morning [76,87,88,89]. 

Chen et al. performed a cross-sectional study that looked into the sleep problems of children with ASD in China [20]. The information in this study is part of the China Multi-Center Preschool Autism Project, and it includes children from 13 cities. Multiple scales and methods were used to collect information from participants. The CSHQ was utilized to obtain information about sleep problems. For autism diagnosis and assessment, the DSM-5, child autism rating scale (CARS), social responsiveness scale (SRS), autism behavior checklist (ABC), and revised children neuropsychological and behavior scale (CNBS-R2016) were used. The number of children with ASD who had sleep disorders was significantly higher than the number of children in the control group with sleep disorders. Scores on the CSHQ were also significantly different between these two groups, with the ASD group being higher, meaning worse sleep problems. Each subscale of the CSHQ had higher scores or prevalence in the ASD group as well. Samanta et al. also demonstrated similar findings, including increased bedtime resistance in children with ASD (and in children with TD, but with smaller percentages). Interestingly, this study examined the most significant sleep problems identified in the study (bedtime resistance, sleep anxiety, sleep onset delay, and daytime sleepiness) in comparison to key features and symptoms of ASD. Bedtime resistance was the subcategory with the highest prevalence among the ASD group, and these children had higher SRS scores. With increased bedtime resistance, children also had lower CARS scores and ABC language scores; however, these relationships warrant further investigation since other subscales did not show the same associations. Among the SRS in this group, social awareness, communication, and cognition were the main functions negatively affected. Another important factor was daytime sleepiness, which was positively correlated with autism symptoms, as these children scored higher on three different scales (ABC, CARS, and SRS). These results further support the idea that sleep problems are worse in children with ASD. Families and caretakers of those with ASD should be engaged in conversations about the sleep patterns and habits of the affected child to aid in the timely management of any sleep abnormalities along with other presenting symptoms of autism [20]. 

In addition to looking at general sleep habits, Berloco et al. specifically examined the occurrence of insomnia [28]. This was a cross-sectional cohort study that aimed to look into the prevalence of insomnia in the pediatric ASD population. Specifically, the Pediatric Sleep Clinical Global Impression Scale was utilized to look at their association with sleep disorders. Using the International Classification of Sleep Disorders, third edition (ICSD-3) criteria, the researchers ascertained that 32% of the 270 participants fell into the category of chronic insomnia. Lastly, parental stress levels were gauged with the Parenting Stress Index–Short Form (PSI-SF). In this study, there was no significant relationship between the cognitive level and sleep problems, as there were no significant differences between high- and low-functioning children with ASD on the Sleep Clinical Global Impression Scale (S-CGI) sections. The S-CGI data also showed that a worse adaptive behavior was associated with increased sleep problems. However, the direction of this correlation is currently unknown; meaning, it is not known if these symptoms cause sleep problems, or if the sleep problems cause these symptoms. Either way, treating both behavioral symptoms and sleep disturbances would be beneficial in children with ASD. With regard to parents, there is a positive relationship between parental stress and both the sleep onset delay score and overall insomnia. It appears that the management and treatment of sleep problems could ease parental stress. Overall, these results point towards the importance of the clinician exploring sleep patterns and habits in individuals with ASD [28]. 

### 8.1. Sleep Disorders and Functional Impairments

These sleep disorders exacerbate the symptoms of ASD; thus, it is important to address them as early as possible. Saletin et al. analyzed the impact of sleep problems on the functional impairment in individuals with ASD to show how screening for such problems is helpful clinically [22]. In order to study the impact of sleep on ASD and vice versa, a large and diverse sample from the ASD population was chosen using the Rhode Island Consortium for Research and Treatment (RI-CART). RI-CART includes individuals of diverse ages from across the state of Rhode Island. In this survey, investigators questioned parents about the presence of current or past sleep problems. To confirm the ASD status of participants, researchers used the autism diagnostic observation schedule, second edition (ADOS-2). Three groups were made within the large grouping of probands: positive based on the original self-report of a community diagnosis and positive with the ADOS-2 (ASD), positive on one or the other (ASD inclusive), and negative on both (non-ASD). The Vineland Adaptive Behavior Scales (VABS) evaluated adaptive skills, with a lower score coordinating with poorer adaptive behavior. Among the skills and behaviors evaluated were life skills, social skills, and communication skills. Social processing issues were gauged with the social responsiveness scale, version 2 (SRS-2). On this test, a higher score is worse. ASD status was found to be positively associated with sleep problems; the ASD group had more sleep problems compared to the ASD-inclusive and non-ASD groups. The ASD group had a 1.58 times higher odds of reporting sleep problems compared to the non-ASD group. As for covariates, the age at enrollment and a multiracial identity predicted higher rates of sleep problems. Looking at data from the VABS, a diagnosis of ASD is related to worse adaptive behavior. Also, individuals with current or past sleep problems had worse functional impairment when compared with individuals who did not report a history of sleep problems. In this case, multiple covariates were significant. Adaptive behavior ratings were worse in those who were older. Participants with caregivers who were educated to at least a high school level were higher functioning than the participants with caregivers having less than a high school education. Also, there were significant findings in the race category. Participants who selected that they “prefer not to respond” had higher adaptive functioning when compared to Caucasian participants. There is a known relationship between confirmed ASD diagnosis and poor functioning. Despite this, there was still a significant relationship between SRS-rated social functioning and sleep problems. On the SRS, female participants had worse scores than male participants. Overall, the data show the importance and usefulness of screening questions for sleep disorders, as they may predict functional outcomes. Children with a confirmed ASD diagnosis have more sleep problems than neurotypical children. By using a sample of individuals all under 21 years of age, this study shows that from a young age, there is an association between sleep and ASD [22]. 

As reported by the study of Saletin et al., disordered sleep is associated with lower functioning [22]. This includes behavior problems and/or more severe symptoms of ASD. Pattison et al. conducted a randomized control trial (RCT) looking at the effects of behavioral sleep interventions with 247 autistic children aged 5–13 [23]. Specifically, they looked at the Sleeping Sound intervention to see if it resulted in fewer sleep problems in autistic children 12 months after randomization. The Sleeping Sound intervention group was compared to a treatment-as-usual (TAU) control group. Individuals in the TAU group were instructed to continue their regular course of care. The last surveys for this study were collected in February 2020, before the COVID-19 pandemic. The children who were randomized to the intervention group were found to have a larger reduction in overall sleep problems by the 12-month follow-up. The Sleeping Sound program resulted in statistically significant differences over 12 months in mean ‘Children’s Sleep Habits Questionnaire’ (CSHQ) scores. In all subscales on the CHSQ, except for sleep-disordered breathing subscale, participants in the intervention group had significant decreases. As for secondary child outcomes, participants in the intervention group scored lower on the SCQ reciprocal social interaction subscale. Parents/caregivers of those in the intervention group scored lower on the Kessler psychological distress scale (K10). Autistic children in the intervention group experienced less significant bedtime resistance, parasomnias, and sleep onset delay, as well as increased sleep duration compared to those in the TAU control group. In general, this study shows that the Sleeping Sound intervention may be helpful for most autistic children [23]. 

### 8.2. Sleep Disorders and Developmental Delays 

In addition to studying ASD, Reynolds et al. also considered other developmental delays and disorders [24]. They conducted a study that was built off another case-control study called Study to Explore Early Development (SEED). In this study, the effect of treatments such as melatonin on the improvement of CSHQ was examined. Overall, children using melatonin had higher mean total scores on the CSHQ than the group who did not use any melatonin. Among the ASD group, there were higher scores on the CSHQ when compared to the general population and other developmental delays without ASD group. These scores were not significantly higher when comparing the developmental delays and disorders group with the ASD group. The data generally demonstrated that children with ASD or developmental delays with ASD had more sleep problems than children in the general population as well as the group of children with developmental delays but without ASD. Future research should therefore look into the link between developmental disorders, such as ASD, and their relation to disordered sleep [24]. 

### 8.3. Awareness Regarding Sleep Disorders

The first step in addressing sleep problems in individuals with ASD includes educating both the patient and the caregiver. This involves sharing information about sleep that is appropriate for the developmental stage of the patient, along with basic behavioral strategies for families to foster optimal sleep conditions [90]. One study found that providing sleep-related information through pamphlets did not enhance the sleep quality in children [91]. However, conducting small group informational sessions proved beneficial in improving both parents’ perceptions of sleep and reducing the time it takes for children to fall asleep [90,91]. Behavioral therapy should begin in early childhood, with adjustments tailored to the child’s age and developmental stage as they grow [92]. Behavioral interventions may encompass strategies such as cognitive–behavioral techniques, extinction protocols, sleep hygiene education, chronotherapy, planned awakenings, sleep restriction, the use of visual aids, stimulus fading, and incentive-based programs. The most effective approach is personalized treatment, beginning with education and behavioral sleep therapies. This method focuses on tailoring a treatment plan to address the unique problems of the individual, recognizing that these issues can vary within this group [90]. If the initial behavioral interventions do not yield success, it is advisable to consider a combined approach of behavioral and pharmacological treatments as a secondary strategy [93]. 

At present, the Food and Drug Administration (FDA) has not approved any medications specifically for treating sleep issues in children with ASD. Nonetheless, many patients utilize medications that are prescribed off-label for this purpose. Approximately 40% of children with ASD are prescribed medications that have demonstrated efficacy in improving sleep in different populations. These include treatments such as melatonin, alpha-agonists, anticonvulsants, antidepressants, atypical antipsychotics, and benzodiazepines to address sleep difficulties [94,95]. Presently, melatonin is the most researched and supported option for its effectiveness in enhancing sleep onset times in individuals with ASD [96].

### 8.4. Adverse Effects of Sleep Disorders on Families

Phadraig and Smyth noted the lack of research about the impact of these ASD-associated sleep problems on the family unit [25]. The goal of their study was to delve into the sleep quality of parents of autistic children to see the effect on family function. Participants were split into two groups: the autism group (parents of autistic children) and the TD group (parents of TD children). The Pittsburgh Sleep Quality Index (PSQI) is a retrospective scale which looks back over one month. Specifically, it pertains to sleep quality and sleep disturbances in both groups. On the CSHQ, autistic children had significantly higher scores than TD children; therefore, autistic children show increased sleeping problems. When parental data were assessed, they found that sleep quality was significantly worse in the autism group when compared to the TD group. Looking at positive relationships, both groups showed that sleep problems in children correlated to worse parental sleep quality. Among both groups, a worse sleep quality was associated with decreased family functioning. Therefore, families in the autism group had a less healthy family function, which was significant compared to the TD group. Matching with previous research in the field, autistic children were more likely to have sleep problems or disturbances than TD children [25]. 

Johnson et al. conducted a randomized clinical trial in children with ASD [26]. The sample was comprised of individuals who participated in a Research Unit on Behavioral Intervention (RUBI) study. Multiple surveys and tests were used to gather data. First, to assess intellectual functioning, researchers used either Stanford–Binet or the Mullen scales. Then, the modified Children’s Sleep Habits Questionnaire modified for ASD (CSHQ-ASD) was completed by the parents of the participants. Next, the aberrant behavior checklist (ABC) was filled out by the parents of the participants. Lastly, the parenting stress index (PSI) was used to assess parental stress. On the ABC, poor sleepers scored higher in all sections except inappropriate speech. The irritability and hyperactivity/noncompliance sections of the ABC had the largest difference among participants in the poor sleep category. Behavior and functioning during the day may therefore be related to poor sleep, as shown by the increase in the irritability and hyperactivity scores that poor sleepers displayed. The other two elevated scales on the ABC included social withdrawal and stereotypy. Participants in the poor sleep category had an increased social disability based on these results. In addition, stereotypy may indicate repetitive behavior often seen with ASD. Significant results were also seen in the parents of children in the poor sleep group. These parents scored significantly higher on the PSI than parents in the good sleeper group. This stress could be caused by a variety of factors, such as general stress from having a child with ASD or stress between the parent and child at bedtime. More research is needed to evaluate parent–child relationships in the context of ASD comorbidities [26]. Many of the studies thus far, if not all, have shown the importance of addressing sleep problems in those with ASD. Samanta et al. discusses the importance of this in a very specific population [97]. They conducted a cross-sectional study that looked at a population of male children aged 2–6 years old with autism in Bhubaneswar, India, to show the need for intervention in these children when sleep problems present themselves. Initially, general lifestyle information was collected about each child, which included parameters such as screen time, exercise per week, birth weight, mother’s age, etc. With regard to sleep, mothers were asked about the recent sleep behavior of their child and then given the CSHQ to fill out. The average mean score on the CSHQ (66.86 +/− 14.77) was above the cutoff for a typical sleep problem group [97]. In addition, many of the subscales on the questionnaire were significantly related. Among the subscales, 95% of participants had a bedtime resistance-related sleep problem within the past month. Overall, a 93% prevalence of sleep problems was found among the participants. These results highlight that adverse sleep problems among male Indian children who have autism are quite common. The relationship between sleep problems and the other factors was studied. In particular, screen time had an inverse relationship with multiple sleep subscales, such as bedtime resistance, as well as the total score on the CSHQ. The light and distraction from electronic devices may have contributed to this outcome. Caffeine intake was positively associated with the sleep subscales of bedtime resistance and parasomnia, and more exercise correlated with fewer sleep problems among certain subscales such as sleep onset delay. Knowing this, exercise could be used as a means of improving sleep problems in this population. Maternal age was inversely related to subscales such as parasomnia, which is a novel finding of this study. Lastly, younger children had a higher prevalence of sleep problems, as shown by the inverse association between age and the night-waking subscale. One limitation of this study is that it was very specific in terms of the location and demographic, so extrapolating these results may not be feasible, although they do seem to correlate well with the findings of other studies discussed here [97]. 

Sleep difficulties in children affect the whole family, with research showing a decrease in the well-being of caregivers. This decline is often due to the sleep debt they accumulate while addressing their child’s sleep problems or health concerns [98]. The burden of caregiving adversely impacts parenting practices and overall family dynamics [99]. Within families of children with ASD, both caregivers and siblings experience higher incidences of sleep difficulties [74,75,77,100,101]. Considering the extensive impact of sleep disorders in a child’s life, their management needs to be flexible to accommodate changes in the patient. While the sleep challenges faced by families raising children with autism are recognized, there is a scarcity of treatments or evidence-based practices designed to support not just the child, but the entire family [99,102,103,104].

### 8.5. Sleep Disorders and Circadian Rhythm

The biological clock, otherwise known as the circadian rhythm, is important in sleep and it may play a role in ASD. A review by Lorsung et al. aimed to delve into the possible connection between these circadian rhythms and ASD [105]. Melatonin, cortisol, and serotonin are the main biomarkers involved in circadian patterning, and their levels may be atypical in those with ASD, contributing to the symptoms of the disorder. Melatonin prompts sleep and has been found to be lower in the ASD population. Low melatonin levels, specifically in children with ASD, were found to be related to disordered sleeping. Cortisol, a stress hormone, was noted to have varying results among studies of individuals with ASD, which may be due to a disordered rhythm of secretion. Individuals with ASD are hypersensitive to stress and experience stress often, which contributes to more variability in cortisol secretion. When older children with ASD were screened, they were seen to have higher overall cortisol levels after a perceived stressful event when compared to neurotypical children, indicating a heightened response to the stress. Serotonin levels are often higher in those with ASD, especially children. Further investigation on the hormonal landscape of ASD is crucial to help uncover more pathogeneses and their effects on sleep [105]. Looking more specifically into circadian rhythms and sleep problems, Martínez-Cayuelas et al. conducted a cross-sectional study that compared sleep problems between an ASD group and a normal intellectual functioning group (TD group) of both children and adolescents [106]. An ambulatory circadian monitoring (ACM) device was used to gather data from participants, It collected information such as the wrist skin temperature, time in movement, etc. Parents also kept a sleep–wake diary. The TAP variable, thermometry, actimetry, and body position were used to gather the sleep and wake periods of participants. Next, awakenings were tracked by movement times that made up “wake bouts.” A “wake bout” was then defined as at least 30 s of wakefulness. Other important values were the TST, which is the number of minutes between sleep onset and sleep offset, sleep-onset latency (SOL), wake time after sleep onset (WASO), time in bed (TIB), and sleep efficiency (SE). The analysis showed that when compared to the control group, the ASD group had a significantly higher TST and SOL, and significantly lower SE. Looking at light exposure, the control group had more; both total light and blue light exposure were greater. Interestingly, the ASD group was found to be more active at night than during the day with regard to motor activity. Differences in the wrist temperature between the ASD group and control group were also found to be significant, especially since body temperature has an impact on the circadian rhythm. ASD groups were found to have less time spent sleeping, worse sleep efficiency, and more latency, which is in line with other studies. Based on these results, the study found that sleep and circadian rhythms were disrupted in children and adolescents with ASD [106]. Early interventions to enhance sleep habits in individuals with ASD would not only benefit their functional capacity but would positively impact the family unit as a whole. 

### 8.6. Treatment Options for Sleep Problems in Autism

In the past three years, intervention studies have included the following: (1) three studies emphasizing the positive effects of physical activity on sleep [107,108,109], (2) two trials observing sleep improvements with the use of extended-release melatonin [110,111], and (3) three studies examining the implementation of individualized functional behavioral interventions to enhance sleep [112,113,114]. These studies on behavioral intervention add to a substantial body of research demonstrating its effectiveness in this population. The investigations into extended-release melatonin are a relatively new field, providing families with an additional, comparatively low-risk method to address sleep onset and maintenance issues, though standard formulations may not resolve all sleep disorders in this group. The three studies focusing on physical activity contribute to an expanding research area highlighting the advantages of physical activity in various health and developmental areas, including sleep.

## 9. Conclusions

Our review summarizes the prevalence of comorbid conditions in ASD, specifically in relation to otolaryngology (Figure 2). Autism and otolaryngology are linked together in many key areas, including hearing loss, CAPD, otitis media, sinusitis, and sleeping disorders. Sleeping problems can affect not only the individual with autism, but also their family’s functioning [25]. Sinusitis and ear infections are both seen at higher rates in those with autism. The pain associated with these diseases may contribute and exacerbate to some of the core symptoms of autism, including self-harming behaviors and hyperacusis, and may be worsened by comorbid language deficits. Hearing problems have a significant prevalence in this patient population. It has been found that the prevalence of hearing loss in individuals diagnosed with autism is 10 times higher than in the general population [34]. CAPD is also common in individuals with autism. These conditions often cause significant impairment to the patient and can be implicated in the core symptoms of autism. This review will help otolaryngologists and other healthcare providers to screen for and promptly recognize these comorbid conditions commonly seen in autistic individuals. The early intervention for these otolaryngology complications will lead to better clinical outcomes, leading to an improved quality of life for individuals diagnosed with ASD and their families/caregivers.

## Figures and Tables

**Table 1:** A categorical scoring approach of functional speech perception skills and expression vocabulary skills (adapted from Eshraghi et al. [38]).

**Speech Perception Categories**	
**Category**	**Perception Criteria**
0	No awareness of environment
1	Awareness, detection, or localization of sounds
2	Identification/recognition of words
3	Identification/recognition of simple (2 word) phrases or commands
4	Understands conversations
**Speech Expression Categories**	
**Category**	**Expression Criteria**
0	No vocalization
1	Some vocalization (consonants, vowels, nasal sounds)
2	Only words
3	Simple phrases and commands
4	Produces sentences

## Data Availability

No new data were created or analyzed in this study. Data sharing is not applicable to this article.

## Abbreviations

ABA	applied behavioral therapy
ABC	autism behavior checklist
ABR	auditory brainstem response
ACM	ambulatory circadian monitoring
ADHD	attention deficit hyperactive disorder
ADOS-2	Autism Diagnostic Observation Schedule, second edition
AEF	auditory evoked field
APD	auditory processing disorder
ASD	autism spectrum disorder
BAEP	brainstem auditory evoked potential
CAP	central auditory processing
CAPD	central auditory processing disorder
CARS	child autism rating scale
CELF-4	Clinical Evaluation of Language Fundamentals, fourth edition
CI	cochlear implant
CNBS-R2016	revised children neuropsychological and behavior scale
CSHQ-ASD	Children’s Sleep Habits Questionnaire modified for ASD
CSHQ	Children’s Sleep Habits Questionnaire
CTOPP	Comprehensive Test of Phonological Processing
dB	decibel
EEG	electroencephalogram
FDA	Food and Drug Administration
GI	gastrointestinal
ICSD-3	International Classification of Sleep Disorders, third edition
IEP	individualized education plan
IM	intramuscular
IQ	intelligence quotient
K10	Kessler psychological distress scale
kHz	kilohertz
MMN	mismatch negativity paradigm
Ms	milliseconds
MTFR	methylenetetrahydrofolate reductase
NMDA	N-methyl-D-aspartate receptor
PE	pressure equalizer
PSI	parenting stress index
PSI-SF	Parenting Stress Index–Short Form
PSQI	Pittsburgh Sleep Quality Index
RCT	randomized control trial
RI-CART	Rhode Island Consortium for Research and Treatment
RUBI	Research Unit on Behavioral Intervention
S-CGI	Sleep Clinical Global Impression Scale
SE	sleep efficiency
SEED	Study to Explore Early Development
SNHL	sensorineural hearing loss
SOL	sleep-onset latency
SRS	social responsiveness scale
SRS-2	social responsiveness scale, version 2
TAU	treatment-as-usual
TD	typically developing
TIB	time in bed
TST	total sleep time
TT	tympanostomy tube
VABS	Vineland Adaptive Behavior Scales
WASO	wake time after sleep onset
